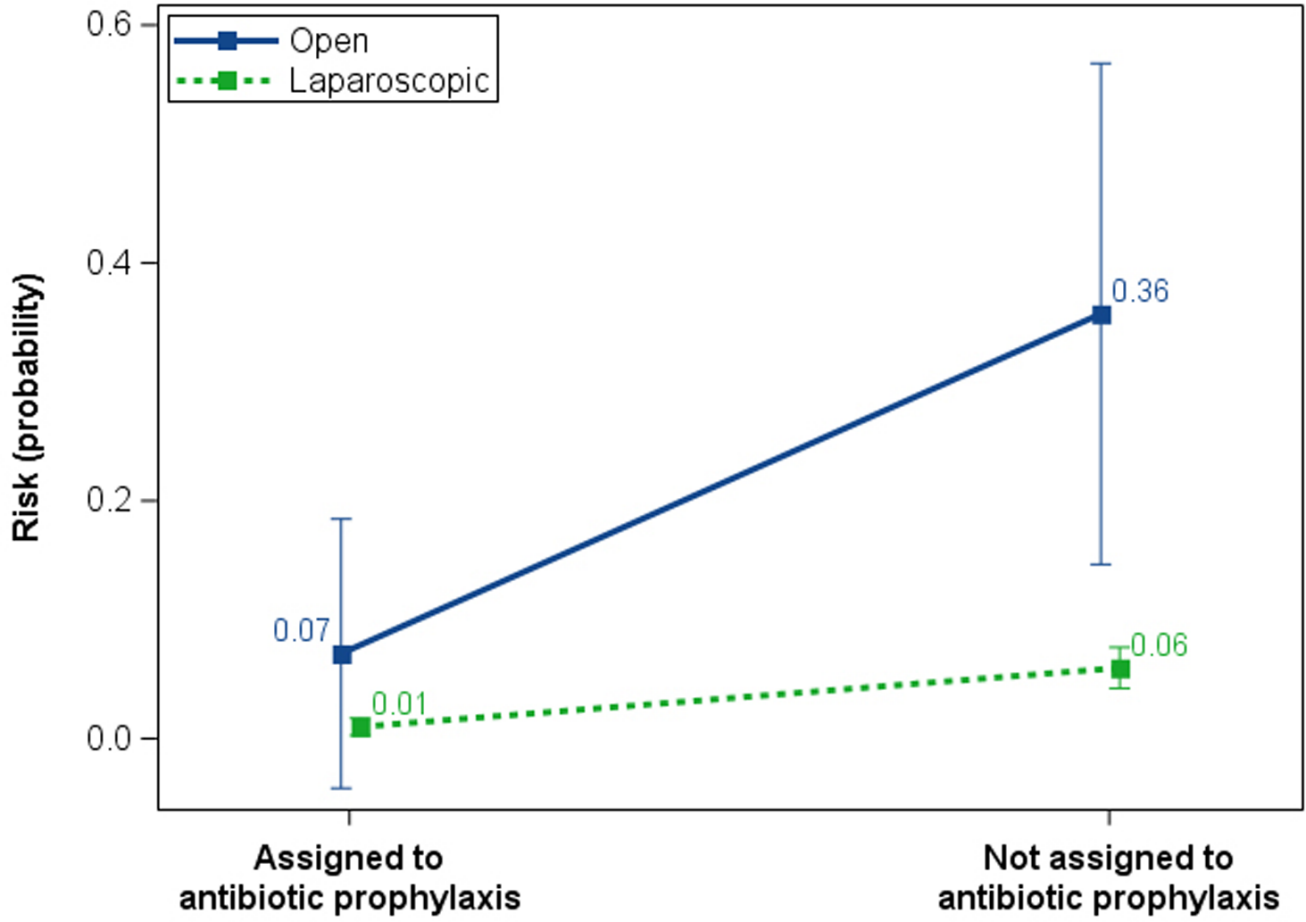# Effect measure modification in an RCT of antibiotic prophylaxis in laparoscopic cholecystectomy: A secondary analysis

**DOI:** 10.1017/ash.2024.314

**Published:** 2024-09-16

**Authors:** Rebecca Zimba

**Affiliations:** CUNY Institute for Implementation Science in Population Health

## Abstract

**Background:** Antimicrobial resistance is a growing public health threat. To alleviate selective pressure which leads to the emergence and proliferation of resistance, and to preserve the utility of treatments where they are needed the most, antimicrobial stewardship programs (ASPs) could consider revising surgical antibacterial prophylaxis protocols for low-risk procedures such as laparoscopic cholecystectomy. **Methods:** Publicly available data was used from a well-powered randomized controlled trial conducted by Matsui and colleagues during 2007-2013 in Japan on the effectiveness of prophylactic antibacterial treatment on reducing post-operative infections following laparoscopic cholecystectomy in low-risk patients. A total of 1037 patients were randomized to receive treatment or no treatment. After randomization, laparoscopic cholecystectomies were converted to open procedures in 28 patients, 14 in each arm, constituting a deviation from the protocol and the administration of additional antibacterial treatment. The original study included both intention-to-treat and per-protocol analyses, finding statistically significant reductions in post-operative infections in the treatment vs no treatment arm (1.2% vs 6.7%, p<0.0001; 1.0% vs 5.9%, p<0.0001, respectively). In the present analysis I assessed the extent to which type of procedure modified the effect of antibacterial prophylaxis on post-operative infection using both additive and multiplicative interaction. Risk and risk differences were estimated using a linear-binomial model and risk ratios were estimated using a log-binomial model. Alpha was set to 0.10. The lowest risk categories for each variable, being assigned to treatment and receiving a laparoscopic procedure, defined the common reference category. **Results:** A 35 percentage point (pp) increase in the risk of post-op infections relative to the reference category was attributable to the joint effects of omitting prophylaxis and conversion to an open procedure (90% CI 14, 56), compared to a 5 pp increase attributable to omission of prophylaxis (90% CI 3, 7) or a 6 pp increase attributable to conversion to an open procedure (90% CI -5, 18) by themselves. The interaction contrast capturedthis super-additive 24 pp increase in risk (90% CI -1, 47). The relative excess risk due to interaction was 23.81 (90% CI -5.56, 53.19), suggesting a departure from additivity as well. **Conclusions:** Patients undergoing open cholecystectomies stand to benefit the most from antibacterial prophylaxis compared to patients who have laparoscopic cholecystectomies. ASPs could consider reducing or eliminating surgical prophylaxis in low-risk procedures such as laparoscopic cholecystectomy to alleviate selection pressure for antibacterial resistant organisms and preserve its effectiveness for people undergoing higher risk procedures.

**Figure:**